# Collaborative Care Versus Consultation Liaison for Patients With Depression or Anxiety Disorders in General Practice in Denmark: 18-Month Follow-Up From the Collabri Flex Trials

**DOI:** 10.1155/da/2909617

**Published:** 2025-06-05

**Authors:** Nadja Kehler Curth, Siv Therese Bogevik Bjørkedal, Carsten Hjorthøj, Ursula Brinck-Claussen, Kirstine Bro Jørgensen, Susanne Rosendal, Anders Bo Bojesen, Merete Nordentoft, Lene Falgaard Eplov

**Affiliations:** ^1^Copenhagen Research Center for Mental Health, Mental Health Center Copenhagen, Copenhagen University Hospital, Mental Health Services—Capital Region of Denmark, Hellerup, Denmark; ^2^Department of Public Health, Section of Epidemiology, University of Copenhagen, Copenhagen, Denmark; ^3^Psychotherapeutic Clinic, Mental Health Center Copenhagen, Copenhagen University Hospital, Mental Health Services—Capital Region of Denmark, Copenhagen, Denmark; ^4^Faculty of Health and Medical Sciences, Institute of Clinical Medicine, Copenhagen, Denmark

**Keywords:** anxiety, care manager, collaborative care, cognitive behavioral therapy, depression, general practice

## Abstract

**Introduction:** To our knowledge, no research has reported long-term follow-up results from studies comparing collaborative care to consultation liaison in general practice. We have earlier reported 6-month follow-up. In this article, we report the 18-month follow-up results from the two Danish Collabri Flex studies.

**Methods:** We compared collaborative care to consultation liaison in two randomized controlled trials for persons with anxiety disorders and depression, respectively. Both interventions sought to improve the quality of depression and anxiety care, but they differed in the extent mental health specialists were involved. As part of the consultation liaison intervention, psychiatrists and care managers could provide advice and suggestions to the general practitioner (GP). In the collaborative care intervention, psychiatrists and care managers could provide advice and suggestions, and care managers could help the GP manage patient care. 18 months after randomization, we collected follow-up data. Outcomes included anxiety symptoms (BAI), depression symptoms (BDI-II), well-being (WHO-5), functional disability (Sheehan Disability Scale), general psychological symptoms (SCL-90-R), and others.

**Results:** In the depression trial, we found a statistically significant difference in depression symptoms between intervention groups at 18-month follow-up, in favor of collaborative care (4.4, 95%CI 2.8–7.0, *p* ≤ 0.001). Many other outcomes showed significant differences between groups, such as anxiety symptoms, functional level, well-being, general psychological symptoms, and self-efficacy. In the anxiety trial, we found no statistically significant difference between groups in anxiety symptoms (1.2, 95%CI −0.3–2.7, *p* ≤ 0.126). In this trial, significant differences between groups were found in outcomes measuring depression symptoms and general psychological symptoms but not in outcomes measuring functional level, well-being or self-efficacy. In both trials, no differences were found between groups on employment/education or sick leave measures. However, the collaborative care group in both trials had fewer contacts with private practicing psychologists during the 18 months follow-up.

**Conclusion:** At 18-month follow-up, we found a statistically significant difference between allocation groups, when measuring depression symptoms in the depression trial but not when measuring anxiety symptoms in the anxiety trial. Further, the collaborative care intervention may have the potential to reduce referrals to private practicing psychologists, a service that is in high demand in Denmark.

**Trial Registration:** ClinicalTrials.gov identifier: NCT03113175 and NCT03113201

## 1. Introduction

Depression and anxiety disorders are common conditions in the general population and problems with access to good quality care exists [1, 2]. Various models, including consultation liaison and collaborative care, have been proposed to improve the management of depression and anxiety disorders in primary health care [3, 4]. The models differ from each other in how they consider the role of the primary care provider and to which extent mental health specialists are involved in the care [4]. In consultation liaison, the primary care provider takes the lead in delivering mental health services to their patients, with help from the mental health specialists (e.g., psychiatrists) who offer support, advise, education, or feedback to the general practitioner (GP), for instance in regard to diagnostic assessments or treatment guidance. Different consultation models exist, and the models can diverge, for example, in how many contacts there are between the primary care provider and the mental health specialist and how many contacts there are between the mental health specialist and the patient [5–7]. Key features of collaborative care are a multiprofessional approach, including a primary care provider and minimum one other professional involved in the care of patients; a structured management plan based on evidence-based guidelines; scheduled follow-up examination(s) of patient progress; and enhanced interprofessional communication mechanisms such as patient-specific feedback or specialist supervision [8, 9]. The research literature about short-term effects of consultation liaison is limited and unclear [6, 10]. The research on longer-term effects is also scarce, and a meta-analysis found no significant depression effects, measured at 12 months or more, when compared to standard treatment [6]. More research has been conducted concerning collaborative care. In a large systematic review and meta-analysis, collaborative care was found effective in reducing anxiety and depression symptoms for both persons with anxiety and depression for up to 24 months, compared to treatment as usual [8]. However, most included studies were carried out in the US and not necessarily comparable to other contexts. A randomized trial in Sweden found significant differences between groups, in favor of collaborative care, in depression symptoms (MADRS-S) at 6 and 12 months but not at 24 months, when compared to usual care [11, 12]. Two studies have compared collaborative care with interventions characterized by consultation liaison, and suggest that in the short term (≤3 months) collaborative care may be more effective than consultation liaison in improving depression outcomes [13, 14]. At 9-month follow-up, one study did not find significant differences between collaborative care and consultation liaison in depression symptoms [14], and to our knowledge, no research has compared the two quality improvement models on longer-term outcomes (12 months or more).

To improve the quality of care for patients with depression and anxiety disorders in primary health care in Denmark, the Collabri Flex study [15] was launched in 2018. The Collabri Flex study investigated the effectiveness of collaborative care compared to consultation liaison. It consisted of two individual-level randomized controlled trials targeting patients with depression and anxiety disorders in general practice. At 6-month follow-up, significant effects of collaborative care were found in both trials for the primary outcomes of anxiety and depression symptoms [16]. After trial commencement, we got the opportunity to conduct an additional follow-up assessment, and this article reports the results of the 18-month follow-up.

## 2. Materials and Methods

### 2.1. Design

The two randomized controlled trials in the Collabri Flex study were pragmatic investigator-initiated, parallel-group superiority trials. One trial had the aim of comparing the effectiveness of collaborative care and consultation liaison for patients with depression, and another trial compared the effectiveness of collaborative care and consultation liaison for patients with anxiety disorders. The two trials had the same overall aim and methods, which are described jointly in this article. Further, detailed descriptions of the recruitment procedures, interventions, randomization, data collection, outcome measures and statistical analysis methods can be found in previous papers [15, 16]. Therefore, a brief outline of the study's methods is reported here.

### 2.2. Setting

According to Danish clinical guidelines, most people with anxiety and depression are expected to be treated in primary mental health care, where the GP should be able to diagnose and provide treatment such as psychoeducation, support, and prescribe medication [17, 18]. GPs participating in supervision with other GPs or with a psychiatrist or a psychologist can be reimbursed for up to seven sessions of talking therapy [19]. In complex cases, or if the GP is uncertain about the diagnosis, the patient can be referred to a psychiatrist, free of charge, or to secondary mental health services [17, 18]. The GP can also refer patients with mild to moderate anxiety and depression to a private psychologist. In such cases, patients above the age of 24 pay 40% of the treatment costs [20, 21]. However, the wait to see a psychiatrist and psychologist is usually long. Lastly, people with anxiety and depression symptoms can also seek treatment outside the public health system. They can see a psychologist or a psychiatrist, at their own expenses, or they may have an insurance that covers a specified number of sessions at a private psychologist.

### 2.3. Participants and Randomization

From January 2018 until September 2019, patients from general practices in the Capital Region of Denmark were recruited. Participating patients were referred to the study by their GP, if the GP assessed a diagnosis of either an anxiety disorder or depression. Participants were included if they were 18 years or older, spoke Danish, provided written consent to participate and fulfilled the criteria for either an ICD-10 diagnosis of depression (F32 or F33), social anxiety disorder (F40.1), panic disorder (F41.0), agoraphobia (F40.0), generalized anxiety disorder (F41.1), obsessive–compulsive disorder (F42) or post-traumatic stress disorder (F43.1). OCD, agoraphobia, and PTSD were added as inclusion diagnoses during the trials. No patients with PTSD were included. Participants were excluded if they needed secondary mental health care, were pregnant, had a diagnosis of dementia, or had an unstable medical condition or preferred treatment by a psychologist instead of the Collabri Flex intervention if they were randomized to this allocation group. Participants were also excluded if they participated in ongoing trials investigating interventions similar to the Collabri-Flex intervention (The Collabri trials and the IBBIS trials [22–24]), to avoid contamination between trials. See previous publications for a more thorough description of the exclusion criteria [15, 16]. As part of the inclusion process, the participant's diagnosis was validated by a care manager using the MINI International Neuropsychiatric Interview [25], adapted with ICD-10-specific questions. All assessments were discussed with either a psychiatrist or a psychologist. Randomization was conducted by a Collabri Flex team member, using a web application developed by the external provider open patient data explorative network (OPEN). The care managers informed the participants and their GPs about the allocation. The allocation sequence was computer generated with variable block sizes hidden from research staff and those performing the randomization during the inclusion period. Randomization was stratified for previous pharmacological or psychological treatment for anxiety or depression, type of anxiety in the anxiety trial, and depression severity in the depression trial. Blinding of GPs, participants, and care managers to the intervention allocation was not possible, but the research team was blinded for allocation groups if they contacted participants at follow-up, e.g., for reminders to fill out questionnaires.

### 2.4. Interventions

#### 2.4.1. Collaborative Care

The Collabri Flex intervention adhered to the collaborative care criteria described earlier and is described more thoroughly elsewhere [15, 16]. The multiprofessional team consisted of the GP, a psychiatrist, and a care manager (a mental health nurse or an occupational therapist with a minimum of 1 year of training in cognitive behavioral therapy (CBT)). Eight care managers employed in the regional mental health services were part of the project and collaborated with 3–5 GPs. Their salaries were paid by project-specific grants from the Danish Ministry of Health. Participants were offered treatment modalities according to disease-specific treatment instructions and stepped care algorithms, providing stepwise intensification of treatment. Treatment could include psychoeducation (independently for 3–4 sessions or as part of CBT), CBT (10–13 sessions, but the length could vary), and medication. Participants were offered disease-specific written information, regular meetings with the care manager to monitor their condition, including the use of structured instruments, reevaluation of the treatment plan, and provision of psychoeducation/and or CBT if this was part of the individualized treatment plan. Participants could invite a next of kin to a care manager session. The sessions with the care manager were carried out biweekly or more frequently, depending on the severity of the disease. Upon initiation of pharmacological treatment, intensified monitoring was implemented with weekly sessions. Reassessments were conducted at a minimum every month, at each escalation in therapy, and upon completion of the treatment regimen. Even though the frequency of monitoring and reassessments was meant to take place at certain intervals, they could differ, due to logistic challenges and cancelations. Sessions between care managers and patients that had a treatment purpose were predominantly face-to-face. Further, care managers offered telephone contacts, for example, between sessions and for booster calls after the treatment had ended. Around half of the care manager sessions were held in general practices. Most other sessions were held in mental health care settings [16]. Psychiatrist supervision of the GPs was provided as preferred by the GP, either ad hoc or regularly. It could take place face-to-face and/or by telephone. Care managers were supervised in providing CBT (by a psychologist specializing in CBT twice a month) and in treatment planning, monitoring, and reevaluation (by a psychiatrist twice a month). Supervision of care managers was mostly carried out face-to-face; however, after 2018, the psychiatrist supervision was changed and took place by telephone. Care managers consulted (most often by telephone) the psychiatrist when conducting baseline inclusion assessments. The psychiatrist and psychologist were employed in the regional mental health services. Their salaries were paid by project-specific grants from the Danish Ministry of Health. Written communication between the care manager, GP, and the psychiatrist was ensured through safe electronic systems.

##### 2.4.1.1. Delivered Treatment

As reported previously, the average length of treatment according to the Collabri Flex model was around 4 months with nine care manager treatment contacts [16]. The content of these contacts depended on the individual treatment plans. Most collaborative care participants had CBT as their initial treatment and around a fifth had their treatment intensified during treatment. A detailed description of treatment delivery in the first 6 months is described elsewhere [16]. During the trial, two fidelity reviews showed that the core model components were generally carried out as intended, and a summary is presented elsewhere [16].

#### 2.4.2. Consultation Liaison

**Figure 1:**
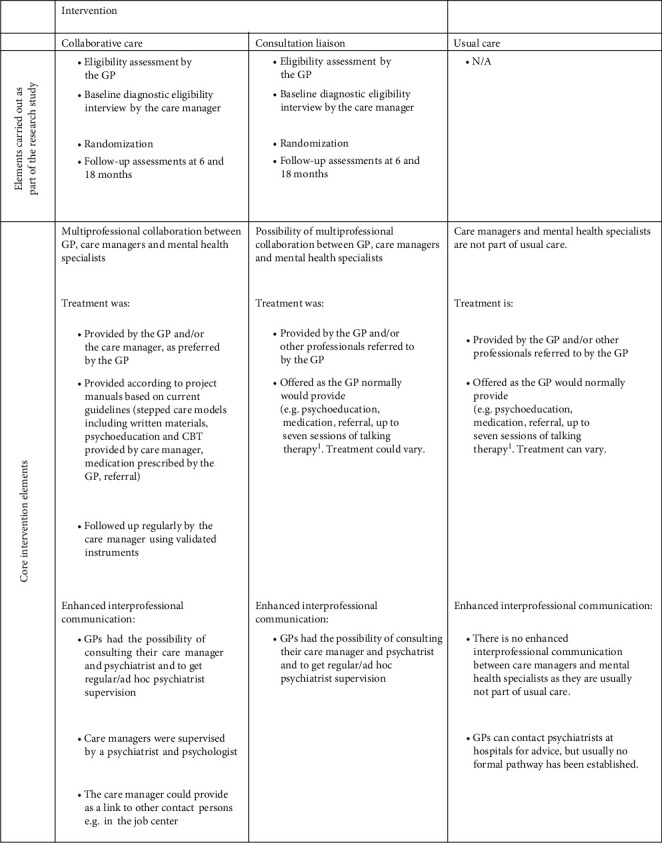
Overview of the core elements of the interventions and usual care in GP practices. ^1^Up to seven talking therapy sessions can be reimbursed to general practitioners (GPs) who participate in supervision with other GPs, a psychiatrist, or a psychologist.

Because collaborative care is an organizational intervention, it would introduce the risk of control group contamination when using individual-level randomization. Therefore, we acknowledged this risk and intentionally added the potentially contaminating elements (assistance in diagnostic assessments, support, and supervision from a mental health specialist) to the control intervention, forming the consultation liaison intervention investigated in this study. The elements could increase the GPs' knowledge and competencies, which could be used when providing care for all patients, not only collaborative care patients. As a consequence of this realization, the consultation liaison intervention offered guidance and advice to the GP by the care managers and/or the psychiatrist, on request from the GPs [16, 15] (see further details for similarities and differences between the collaborative care intervention and the consultation liaison intervention in Figure 1). The GP had the overall responsibility of treatment and provided treatment-as-usual, for example, guided by recommendations from the Danish College of General Practitioners and the Danish Health Authority. Treatment could, for example, include support, talking therapy, psychoeducation, medication, or referral to a private psychiatrist, a private psychologist, or mental health services [17, 18]. Patients allocated to the consultation liaison group had no contact with care managers after randomization.

### 2.5. Outcomes

All outcomes reported in this article are exploratory outcomes. The primary outcomes of these trials were defined at 6 months as anxiety symptoms measured with Beck Anxiety Inventory (BAI) [26] in the anxiety trial and depression symptoms measured with Beck Depression Inventory II (BDI II) [27] in the depression trial. Primary, secondary, and exploratory outcomes measured at the 6-month follow-up are reported elsewhere [15]. Self-reported exploratory outcomes measured at 18-month follow-up reported in this article for both RCTs are based on differences between groups (endpoint comparisons) in:

• Depression symptoms (BDI-II).
• Anxiety symptoms (BAI).
• General psychological symptoms (Symptom scale, SCL-90-R) [28].
• General well-being (WHO-5 Well-being Index) [29].
• Functional level (Sheehan Disability Scale, SDS) [30].
• Self-efficacy (subscale from IPQ-R about personal control and subscales from the Chronic Disease Self-Efficacy Scales: *Control/Manage Depression Scale* and *Obtain Help from Community*, *Family*, *Friends Scale*) [31, 32].
• Health status (EuroQol Five Dimensions Questionnaire, EQ-5D-3L) [33].
• Symptom remission based on BAI in the anxiety trial and BDI-II in the depression trial.

Register-based exploratory outcomes include:

• Outpatient mental health contacts.
• Proportion on sick leave benefits at follow-up and number of weeks on sick leave benefits.
• Proportion in employment/education at follow-up and number of weeks in employment/education.

### 2.6. Other Data Collected

Contacts with a GP, a private psychiatrist, and a private psychologist were also reported. This information is retrieved from the health insurance register which provides information on contacts that are subsidized by the public health insurance. We did not collect data about medication from Danish registers.

### 2.7. Statistical Analysis

All exploratory outcomes at 18-month follow-up were described in the online registration of the trials. However, the outcomes were added after trial commencement, as we got the opportunity to conduct a second follow-up examination after the trial had begun. A detailed statistical analysis plan was developed prior to analyses and deviations from this are described in the Supporting Information (Box S1). Researchers were not blinded during the statistical analyses as the allocation groups were revealed in connection to the 6-month follow-up reporting. Sample size calculations based on the primary outcomes and power calculation of the secondary outcomes were conducted before the trial was initiated [15]. A clinically relevant difference between allocation groups was set to 4 points on the primary outcomes (BAI and BDI-II). The targeted sample sizes were reached, and analyses were performed according to the statistical principle “intention-to-treat”. The analyses using questionnaire data were based on all included participants and missing end-point values were imputed using multiple multivariate normal regression imputations in chained equations, except for the one dichotomous outcome of remission, where we used logistic regression. The continuous questionnaire-based outcomes were analyzed using analysis of variance (ANCOVA), and dichotomous outcomes were analyzed using logistic regression. Count outcomes were analyzed using Poisson regression with nonparametric bootstrapped confidence intervals. All analyses were adjusted for stratification variables and the outcome-specific baseline value. Sensitivity analyses and subgroup analyses were also performed (see further details in Box S1). Analyses were performed in R version 3.6.1.

## 3. Results

In total, 691 participants were recruited from 29 GPs. Among those, 389 persons participated in the depression trial (193 in the consultation liaison group and 196 in the collaborative care group) and 302 persons participated in the anxiety trial (151 in each allocation group). Participant characteristics are presented in Table S1.

**Figure 2:**
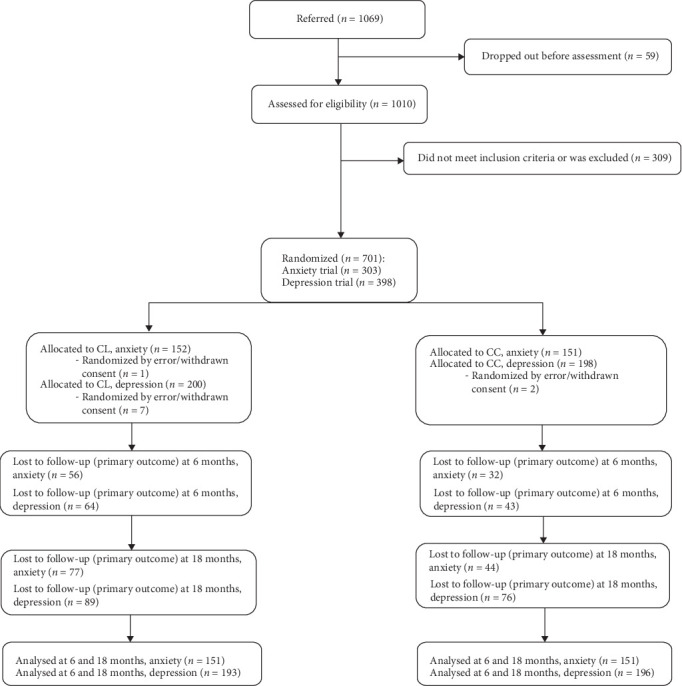
Flow chart.

At 18-month follow-up, a total of 224 in the depression trial filled out the BDI-II (primary outcome) and a total of 181 filled out the BAI (primary outcome) in the anxiety trial. These numbers are corresponding to a response rate of 58% and 60%, respectively (see Figure 2). In the following, results from the depression trial and the anxiety trial are reported separately.

### 3.1. The Depression Trial

**Figure 3:**
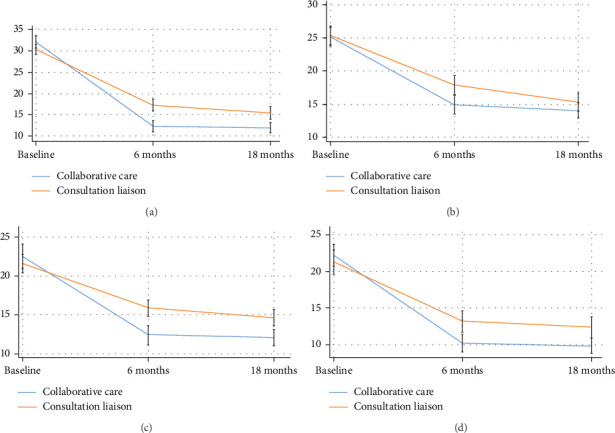
Changes in BAI and BDI-II in the anxiety trial and the depression trial. (a) Changes in BDI-II from baseline to 18-month follow-up in the Collabri Flex depression trial. (b) Changes in BAI from baseline to 18-month follow-up in the Collabri Flex anxiety trial. (c) Changes in BAI from baseline to 18-month follow-up in the Collabri Flex depression trial. (d) Changes in BDI-II from baseline to 18-month follow-up in the Collabri Flex anxiety trial. Both BAI and BDI-II scales goes from 0 to 63 and not from 5 to 25, 30 or 35 as depicted. FU, follow-up.

In the depression trial, there was a drop in depression symptoms (BDI-II) in both allocation groups at both follow-up time points (6 and 18 months), and a statistically significant difference between allocation groups was found at 18-month follow-up (4.4, 95%CI 2.8–6.1, *p* ≤ 0.001) (Figure 3a, Table 1). The between-group difference corresponded to an effect size of Cohen's *d* = 0.37, favoring the collaborative care group. The same tendency was seen for anxiety symptoms (BAI) (Figure 3b, Table 1).

There were statistically significant differences between allocation groups for all other self-reported outcomes including measures of well-being, self-efficacy, functional level, and health status (Table 1). We found no difference between allocation groups on register-based outcomes including outpatient contacts and measures of sick leave and employment/education. Neither did we find differences between allocation groups on measures of mental health care delivery in primary care, except for psychologist contacts (Table S2a). Here, we found a rate ratio of 7.35 (95%CI 4.05–19.97), with the consultation liaison groups having more contacts. Subgroup analyses showed that the between-group difference in depression symptoms was statistically significant for participants with moderate and severe depression, participants with and without previous treatment, and for participants regardless of scores on Standardized Assessment of Personality: Abbreviated Scale (SAPAS) (dichotomized variable, where SAPAS > 2 indicated positive screening for personality disorder). The confidence intervals for the estimated treatment effects in the subgroups indicated no significant heterogeneity across subgroups. The only exception was for patients with mild depression at baseline who had a substantially lower estimated treatment effect than the moderate and severe subgroups. Results from the sensitivity analyses showed no substantial differences from the main analysis. Results from the subgroup analyses and sensitivity analyses can be found in Tables S3 and S4.

### 3.2. The Anxiety Trial

In the anxiety trial, there was a drop in anxiety symptoms (BAI) in both allocation groups at both follow-up time points (6 and 18 months), and a statistically significant difference between allocation groups, in favor of collaborative care, was found at the 6-month follow-up but not at 18-month follow-up (1.2, 95%CI −0.3–2.7, *p* ≤ 0.126) (Figure 3c and Table 2). For depression symptoms, a significant difference between allocation groups, in favor of collaborative care, was found at 18-month follow-up (2.8, 95%CI 1.2–4.4, *p* ≤ 0.001), corresponding to an effect size of Cohen's *d* = 0.32 (Figure 3d and Table 2).

All other self-reported and register-based outcomes, except general symptoms (*p*=0.045), did not show statistically significant differences between allocation groups (Table 2). Neither did we find differences between allocation groups on measures describing mental health care delivery in primary care, except for psychologist contacts (Table S2a). Here, we found a rate ratio of 3.04 (95%CI 1.37–9.52), with more contacts in the consultation liaison group.

Results from the subgroup analyses and sensitivity analyses can be found in Tables S3 and S4. Stratifying participants in terms of primary anxiety diagnosis, previous treatment (yes/no), and a screening for personality disorder/dichotomized variable) did not reveal any statistically significant differences between groups in >anxiety symptoms (Table S4). The confidence intervals for the estimated treatment effects in the subgroups indicated no significant heterogeneity across subgroups. In the sensitivity analyses, we found significant differences between groups in the best- and worst-case scenarios when replacing missing data. However, we consider these analyses as extreme case analyses, and only in the best-case scenario, the difference was in favor of the consultation liaison group.

## 4. Discussion

### 4.1. Summary of Main Findings

Both allocation groups in both trials reduced their anxiety and depression symptoms from baseline to 18-month follow-up. However, the reductions accelerated more rapidly in the collaborative care groups than in the consultation liaison group (Figure 3a–d). At 18-month follow-up, there was still a statistically significant difference between allocation groups, in favor of collaborative care, when measuring depression symptoms in the depression trial but not when measuring anxiety symptoms in the anxiety trial.

### 4.2. Suggested Explanations for Differences Between Trial Results

There could be different explanations for why we find a difference between intervention groups, in favor of collaborative care, in the depression trial (concerning depression symptoms) and do not find a difference between intervention groups in the anxiety trial (concerning anxiety symptoms). Even though analyses suggest no significant heterogeneity across subgroups, several different anxiety diagnoses were included in the anxiety trial and each group was fairly small, with social anxiety disorder and OCD being less represented. This could hypothetically mean that the effects were more or less pronounced for some subgroups, even though we were not able to detect this. Further, the effect size at the 6-month follow-up was smaller for anxiety symptoms in the anxiety trial than for depression symptoms in the depression trial. Thus, after 18 months it could be expected that the differences between intervention groups would have decreased in both trials and thus, with the difference being even smaller in the anxiety trial at 18 months than in the depression trial. Further, even though anxiety disorders and depression share many symptoms and characteristics, they are usually thought to have different prognoses and different disease patterns [34, 35]. These differences could have been reflected in the 18-month symptom levels. Even though there were differences between results in the anxiety and depression trials, we found the same tendency of reduced use of psychologists in the collaborative care groups compared to the consultation liaison groups. We suggest that a reason for this could be, that the collaborative care intervention in many instances was able to meet the patients' need for a psychological intervention and therefore had the potential to reduce psychologist contacts.

### 4.3. A Health Service Perspective on Findings

In terms of number of GP contacts, introducing a care manager as part of the Collabri Flex team, did not lead to statistically significant differences between intervention groups in either trial. There may be several interpretations of the lack of differences in GP contacts between intervention groups. One is that the communication with the mental health specialists in both groups may have empowered the GP to handle the patients mental health concerns and improve the quality of care without requiring additional consultation. Another interpretation of the lack of differences may pertain to the heavy workload many GPs face, and some GPs did perhaps not have the time or the capacity to provide additional consultations. Also, many GPs are trained as generalists and may have, despite the enhanced communication with the mental health specialists, not felt equipped to provide additional services, e.g., talking therapies. Instead, the GPs acted as referral points to other mental health services, which can explain the differences between intervention groups in contact with private psychologists. Notably, it is unclear whether or how the two intervention models led to changes in GP contacts. Including a control group with usual care in comparison to the intervention groups would have illuminated this.

From the presented tables, we have no information about the quality of care, of the GP consultations. For example, the content, complexity, and time spent in the consultations could have been different according to the patient's treatment group. Qualitative research could have helped illuminate this. There were, however, fewer contacts with outpatient mental health services, private psychiatrists, and psychologists in the collaborative care groups, even though differences were only statistically significant regarding psychologist contacts. Thus, collaborative care to a larger extent kept treatment management within the context of general practice.

Having trained nurses or equivalents as care managers, offering psychotherapy in general practice, instead of referring patients to private psychologists may offer several benefits. The care managers can provide immediate or more timely access to mental health care. This is particularly important considering the, often long, wait times for appointments with private psychologists. Consequently, patients can receive support without delay, which is crucial in managing depression and anxiety effectively. Allocating resources to employ care managers in general practices could increase the accessibility of psychotherapy to a larger population and address the existing treatment gap for anxiety and depression disorders [36]. Further, integrating care managers into general practice encourages a team-based approach to patient care, where GPs and nurses or other healthcare professionals collaborate to provide comprehensive support. This approach may strengthen the continuity and quality of care, ensure that patients' needs are met in a coordinated manner, and enhance a holistic approach to address patients‘ physical and mental health. Providing psychotherapy to patients in primary care may also be more cost-effective than referral to a private psychologist. Results from a planned health economic study of the collaborative care model and the consultation liaison model will provide information on the cost-effectiveness of the two treatment models.

### 4.4. Findings in Relation to Other Research

Our main result from the depression trial that collaborative care was more effective than consultation liaison 18 months after baseline (SMD = 0.37), is comparable to results from studies investigating longer-term effects of collaborative care. Two meta-analyses found effect sizes (SMD) ranging between 0.21 (12 months and over) and 0.35 (13–24 months) on depression outcomes [8, 37]. In contrast, a recent Swedish study found effects of collaborative care at 6 and 12 months but not at 24 months [11]. However, this intervention differed from the Collabri Flex depression trial in some ways. For example, most care manager contacts with patients were telephone-based, and care managers did not provide manual-based psychotherapy as an integral part of the intervention [12]. Different intervention elements, carried out within the collaborative care framework, could have contributed to the effects of the Collabri Flex intervention 18 months after randomization. For example, the early onset of evidence-based treatment in a primary care setting could have prevented further deterioration in symptom levels before treatment initiation [38]. Also, the focus on self-efficacy, self-management, and problem-solving skills learned through CBT and provided by closely supervised practitioners, could have prevented relapse or recurrence of symptoms and/or aided individuals in their ongoing process of recovery [39, 40]. However, these suggestions of explanatory components are only speculations, as to our knowledge, research has not investigated, which collaborative care intervention elements are linked to longer-term effects. Further, the nature of collaborative care, being a complex intervention, also makes it difficult to point out isolated effectful elements of the intervention. A process evaluation could have helped us explore, in more depth, the mechanisms of change in this specific collaborative care model.

The previous research concerning collaborative care for populations with anxiety disorders is much sparser than for depression and in particular concerning follow-up studies exceeding 12 months. Previous trials have reported effect sizes for anxiety outcomes ranging between 0.18 (18-month follow-up) and 0.30 (24-month follow-up) [41, 42]. A reason why we do not find a statistically significant effect of collaborative care on anxiety symptoms at 18-month follow-up could be due to a larger part of the consultation liaison population having received more specialized care at this time point (as opposed to at 6 months) with a private psychiatrist or psychologist, and therefore were doing better, making the between-group differences smaller. However, we have not investigated whether the increase in care was statistically significant. Further, we have no information about potential contacts to psychologists, psychiatrists, or mental health care providers who are not part of the Public Health Insurance. If participants in the consultation liaison group have visited such professionals to a high degree, this could also have impacted positively on their symptom levels.

### 4.5. Strengths and Limitations

These RCTs add to the limited collaborative care research on anxiety disorders, research comparing collaborative care with consultation liaison and research investigating long-term effects of these models. It is a strength, that the methods used in the studies follow standards for conducting randomized controlled trials and that the intervention content is informed by learnings from the previous Collabri trials [15, 22], which could have prevented initial implementation challenges. We do not know from these trials, whether collaborative care is superior to treatment-as-usual in a Danish setting, as the control group included the possibility for GPs to consult and liaise with the Collabri Flex team. The involvement of the care manager in the inclusion of participants, the use of MINI interviews and discussion of the diagnosis with the psychiatrists or psychologist, may have improved the diagnostic accuracy, which is considered as crucial in the management of depression and anxiety, as it informs the provision of adequate treatment [43, 44]. Diagnostic assessment can be an element in both collaborative care and consultation liaison [6, 8]. In this study, diagnostic assessment and validation were done for research purposes, but can also have impacted the subsequent quality of treatment provided to participants in both intervention arms.

Even though sub-group analyses did not reveal any differences in effects between the different anxiety diagnoses, it could have impacted on our main results for the anxiety trial, that the anxiety group was quite heterogeneous in terms of diagnoses. We included several anxiety diagnoses, including OCD, which, to our knowledge, no other collaborative care studies have been done before. The change of inclusion criteria after trial initiation, leading to OCD, PTSD, and agoraphobia also being inclusion diagnoses, may have increased the risk of a heterogeneous study population in the anxiety trial, and consequently, heterogeneity of treatment effects, making it difficult to determine whether collaborative care is effective for all anxiety conditions or a subset of anxiety disorders, for instance, panic disorders. However, only 17 participants with OCD and no participants with PTSD were included in the study. Participants with agoraphobia were pooled with the group of participants with panic disorders. Thus, we deem that the change of inclusion criteria in the anxiety trial did not further increase the risk of heterogeneity of treatment effects.

A limitation of this study is that we did not collect information from the Danish registries regarding participants' medical treatment. Thus, we cannot investigate whether between-group differences in depression symptoms at 18 months follow-up can be attributed to the fact that more participants in the collaborative care group were treated with antidepressant medication, compared to those in the consultation liaison group.

The follow-up rates were 58% in the depression trial and 60% in the anxiety trial, which can be expected in longer-term follow-up studies. They were lower for the consultation liaison groups than for the collaborative care groups, which could have introduced the risk of attrition bias. However, the questionnaire-based analyses use multiple imputations, aiming to compensate for this, and the main results are supported by results from sensitivity analyses using observed data only. Health economic analyses are planned. These will add to the further interpretation of results.

## 5. Conclusion

The Collabri Flex intervention aimed to ensure the provision and quality of evidence-based treatment for persons with anxiety and depression in a Danish primary care setting. At 18-month follow-up, we found a statistically significant difference between allocation groups, in favor of collaborative care, when measuring depression symptoms in the depression trial but not when measuring anxiety symptoms in the anxiety trial. At this timepoint, the collaborative care groups in both trials had significantly fewer contacts with private psychologists than did the consultation liaison groups. Thus, the intervention may also have the potential to reduce referrals to a service that is in high demand in Denmark.

## Figures and Tables

**Table 1:** Outcomes at 18-month follow-up in the Collabri Flex depression trial (*N* = 389).

	Collaborative care (*N* = 196)		Consultation liaison (*N* = 193)				
Outcome measures	Mean (95% CI)		Mean (95% CI)		Difference (95% CI)	*p*	Cohen's *d*
BDI-II ↓	12.3 (11.2, 13.5)		15.8 (14.3, 17.2)		4.4 (2.8, 6.1)	≤0.001	0.37
BAI ↓	12.1 (11.0, 13.1)		14.6 (13.5, 15.7)		2.9 (1.7, 4.2)	≤0.001	0.32
SDS ↓	8.6 (7.8, 9.4)		11.2 (10.2, 12.1)		2.7 (1.6, 3.9)	≤0.001	0.41
WHO-5 ↑	55.0 (52.2, 57.8)		46.1 (43.2, 49.1)		−9.7 (−5.9, −13.5)	≤0.001	0.43
SCL-90-R ↓ ^a^	63.7 (58.0, 69.4)		77.8 (71.0, 84.7)		17.5 (10.1, 25.0)	≤0.001	0.32
Self-efficacy (symptoms) ↑ ^b^	6.5 (6.2, 6.8)		6.0 (5.7, 6.3)		−0.6 (−0.9, −0.3)	≤0.001	0.25
Self-efficacy (obtain help) ↑ ^c^	6.5 (6.2, 6.8)		6.1 (5.8, 6.4)		−0.5 (−0.8, −0.2)	0.004	0.18
EQ-5D-3L ↑	0.8 (0.8, 0.8)		0.7 (0.7, 0.8)		−0.1 (−0.1, −0.1)	≤0.001	0.49
Personal control ↑ ^d^	23.3 (22.7, 23.9)		22.3 (21.8, 22.8)		−1.1 (−1.7, −0.5)	≤0.001	0.27

	***N* (%)**		***N* (%)**		**OR (95% CI)**	***p***	

In remission (BDI-II) (%)^e^	120 (61.4)		95 (49.1)		0.50 (0.29, 0.87)	0.015	
In employment/education (%)	114 (58.2)		107 (55.4)		0.97 (0.63–1.50)	0.905	
Receiving sick leave benefits (%)	13 (6.6)		14 (7.3)		1.10 (0.50–2.42)	0.807	

	***N***	**R (95% CI)**	*N*	**R (95% CI)**	**RR (95% CI)**	***p***	

Weeks, employment/education	9173	46.8 (42.3, 51.0)	8310	43.1 (38.1, 47.6)	0.97 (0.86–1.08)	0.626	
Weeks, sick leave benefits	1713	8.7 (6.4, 11.4)	1651	8.6 (6.3, 11.1)	0.95 (0.63–1.38)	0.599	
Contacts, psychiatric outpatient services	442	2.3 (1.2, 3.6)	550	2.9 (1.6, 4.5)	1.26 (0.61–2.84)	0.343	

*Note:* For questionnaire data, estimates are based on imputed data adjusted for baseline values and stratification variables. The rate is the number of events per person per 18 months.

Abbreviations: BAI, Beck Anxiety Inventory; BDI-II, Beck Depression Inventory-II; EQ-5D-3L, EuroQol Five Dimensions Questionnaire with three levels; R, rate; RR, rate ratio; SCL-90-R, Symptom Checklist-90-Revised; SDS, Sheehan Disability Scale; WHO-5, World Health Organization-5 Well-Being Index.

↓ = Lower scores indicate a better outcome. For example, lower scores of BDI-II/BAI indicate lower symptom scores.

↑ = Higher scores indicate a better outcome. For example, higher scores of WHO-5 indicate higher well-being.

^a^The SCL-90-R has a reference period of 2 weeks instead of 1 week.

^b^Subscale *Control/Manage Depression* from the Chronic Disease Self-Efficacy Scales.

^c^Subscale *Obtain Help from Community*, *Family*, *Friends* from the Chronic Disease Self-Efficacy Scales.

^d^ *S*ubscale *Personal control* from the Illness Perception Questionnaire-Revised (IPQ-R).

^e^Remission was defined by a score of 13 or less in BDI-II.

**Table 2:** Outcomes at 18-month follow-up in the Collabri Flex anxiety trial (*N* = 302).

	Collaborative care (*N* = 151)		Consultation liaison (*N* = 151)				
Outcome measures	Mean (95% CI)		Mean (95% CI)		Difference	*p*	Cohen's *d*
BDI-II ↓	9.8 (8.8, 10.9)		12.4 (10.9, 13.8)		2.8 (1.2, 4.4)	≤0.001	0.32
BAI ↓	14.0 (12.9, 15.2)		15.3 (13.9, 16.7)		1.2 (−0.3, 2.7)	0.126	0.16
SDS ↓	8.5 (7.4, 9.5)		8.6 (7.6, 9.5)		0.3 (−1.0, 1.5)	0.688	0.01
WHO-5 ↑	54.1 (51.0, 57.2)		56.4 (53.3, 59.4)		1.9 (2.3, 6.0)	0.377	0.12
SCL-90-R ↓ ^a^	63.1 (56.1, 70.1)		69.0 (61.2, 76.8)		8.0 (0.2, 15.8)	0.045	0.13
Self-efficacy (symptoms) ↑ ^b^	6.9 (6.6, 7.2)		6.6 (6.4, 6.9)		−0.3 (−0.6, 0.1)	0.128	0.15
Self-efficacy (obtain help) ↑ ^c^	7.0 (6.6, 7.3)		7.0 (6.7, 7.3)		0.0 (−0.3, 0.4)	0.803	0.04
EQ-5D-3L ↑	0.8 (0.8, 0.8)		0.8 (0.8, 0.8)		−0.0 (−0.04, 0.03)	0.877	0.03
Personal control ↑ ^d^	23.9 (23.3, 24.4)		23.1 (22.6, 23.6)		−0.6 (−1.26, 0.06)	0.074	0.25

	***N* (%)**		***N* (%)**		**OR (95% CI)**	***p***	

In remission (BAI) (%) ^e^	47 (31.4)		50 (33.0)		1.10 (0.58, 2.10)	0.768	
In employment/education (%)	102 (67.5)		106 (70.2)		0.99 (0.56, 1.73)	0.960	
Receiving sick leave benefits (%)	NA		NA		NA	NA	

	***N***	**R (95% CI)**	***N***	**R (95% CI)**	**RR (95% CI)**	***p***	

Weeks, employment/education	7916	52.4 (47.1, 57.6)	7946	52.6 (47.6, 57.6)	0.93 (0.84, 1.02)	0.881	
Weeks, sick leave benefits	724	4.8 (3.0, 6.9)	675	4.5 (2.7, 6.4)	1.04 (0.57, 1.91)	0.460	
Contacts, psychiatric outpatient services	295	2.0 (1.0, 3.1)	496	3.3 (1.9, 5.0)	1.76 (0.81, 4.02)	0.153	

*Note:* For questionnaire data, estimates are based on imputed data adjusted for baseline values and stratification variables. The rate is the number of events per person per 18 months.

Abbreviations: BAI, Beck Anxiety Inventory; BDI-II, Beck Depression Inventory-II; EQ-5D-3L, EuroQol Five Dimensions Questionnaire with Three Levels; R, Rate; RR, Rate ratio; SCL-90-R, Symptom Checklist-90-Revised; SDS, Sheehan Disability Scale; WHO-5, World Health Organization-5 Well-Being Index.

↓ = Lower scores indicate a better outcome. For example, lower scores of BDI-II/BAI indicate lower symptom scores

↑ = Higher scores indicate a better outcome. For example, higher scores of WHO-5 indicate higher well-being.

^a^The SCL-90-R has a reference period of 2 weeks instead of 1 week.

^b^Subscale *Control/Manage Depression* from the Chronic Disease Self-Efficacy Scales.

^c^Subscale *Obtain Help from Community*, *Family*, *Friends* from the Chronic Disease Self-Efficacy Scales.

^d^ *S*ubscale *Personal control* from the Illness Perception Questionnaire-Revised (IPQ-R).

^e^Remission was defined by a score of 9 or less in BAI.

## Data Availability

Any requests for data sharing should be directed to the last author. Data that are shared upon reasonable request will be anonymized.

## Supporting Information

Supporting Information Box S1: Specifications of statistical methods, Table S1. Baseline characteristics for the Collabri Flex anxiety trial (*N* = 302) and the Collabri Flex depression trial (*N* = 389), Table S2a. Contacts between primary care providers and participants during 18 months in the Collabri Flex anxiety trial (*N* = 302) and the Collabri Flex depression trial (*N* = 389), Table S2b. Proportion of participants having contacts with a private psychologist, private psychiatrist or having talking therapy with a GP during 18-month follow-up in the Collabri Flex depression trial (*N* = 389) and the Collabri Flex anxiety trial (*N* = 302), Table S3. Sensitivity analyses at 18-month follow-up in the Collabri Flex depression trial (*N* = 389) and the Collabri Flex anxiety trial (*N* = 302), Table S4. Subgroup analyses at 18-month follow-up in the Collabri Flex depression trial (*N* = 389) and the Collabri Flex anxiety trial (*N* = 302).
