# Supplementary material for: Collaborative Care Versus Consultation Liaison for Patients With Depression or Anxiety Disorders in General Practice in Denmark: 18-Month Follow-Up From the Collabri Flex Trials
Source: Depress Anxiety. 2025 Jun 5;2025:2909617. doi: 10.1155/da/2909617 (PMC12162161; doi:10.1155/da/2909617)
Supplement: Supporting Information — Box S1: Specifications of statistical methods, Table S1. Baseline characteristics for the Collabri Flex anxiety trial (N = 302) and the Collabri Flex depression trial (N = 389), Table S2a. Contacts between primary care providers and participants during 18 months in the Collabri Flex anxiety trial (N = 302) and the Collabri Flex depression trial (N = 389), Table S2b. Proportion of participants having contacts with a private psychologist, private psychiatrist or having talking therapy with a GP during 18-month follow-up in the Collabri Flex depression trial (N = 389) and the Collabri Flex anxiety trial (N = 302), Table S3. Sensitivity analyses at 18-month follow-up in the Collabri Flex depression trial (N = 389) and the Collabri Flex anxiety trial (N = 302), Table S4. Subgroup analyses at 18-month follow-up in the Collabri Flex depression trial (N = 389) and the Collabri Flex anxiety trial (N = 302). [file 2909617.f1.docx]

**Supplementary file**

**Box S1**

| ***Specifications of statistical methods***  ***Analyses not specified in the online protocol registration but specified in the statistical analysis plan (SAP)***  Count outcomes should have been analysed using negative binomial regression, but the model did not fit well and Poisson regression with non-parametric bootstrapped confidence intervals was used instead. This model specification was also used analysing count data on GP, psychologist and psychiatrist contacts.  Effect sizes were not only estimated for the primary outcomes as specified, but for all the self-assessed outcomes. Effect sizes were estimated by dividing the mean difference by the pooled standard deviation at follow-up. Calculation of remission in the anxiety trial was based on a cut off score of 9 or less and in the depression study remission rate was based on a cut off score of 13 or less.  Three types of sensitivity analyses were performed with 1) observed data, 2) adjustment for variables with baseline differences, and 3) with imputations of missing values representing “worst” and “best” case of missingness. Here, we made two analyses: one where we replaced missing data with high mean values (representing “worst case”, defined as 90th percentile of the observed mean value) and one where we replaced missing data with low mean values (representing “best case”, defined as 10^th^ percentile of the observed mean value). The analyses were specified in the SAP, but the follow-up time was not specified.  Subgroup analyses were carried out using the same ANCOVA model specification as in the primary analyses, but with treatment effects estimated separately for each subgroup. Analyses were carried out for former pharmacological/psychological treatment, anxiety type (in the anxiety trial) and illness severity (in the depression trial) and screening positive for personality disorder (indicated by a cut off> 2 in Standardised Assessment of Personality – Abbreviated Scale, SAPAS) (1). The analyses were specified in the SAP, but the follow-up time was not specified.  ***Analyses not specified in the online protocol registration or the statistical analysis plan***  Analyses presenting proportions having contacts with a health care provider were analysed using logistic regression adjusted for strata and a binary indicator for whether there had been at least one contact 18 months prior to inclusion.  The outcome measure sick leave was not specified in the online registration, but later defined in the protocol and SAP as proportion on sick leave benefits at follow-up and number of weeks on sick leave benefits  The outcome employment was not specified in the online registration, but later defined in the protocol and SAP as proportion in employment/education at follow-up and number of weeks in employment/education  The dichotomous outcome of remission was imputed using logistic regression. This method was not specified in the SAP. |
| --- |

**Table S1. Baseline characteristics for the Collabri Flex anxiety trial (N=302) and the Collabri Flex depression trial (N=389).**

|  | **Anxiety trial** | | **Depression trial** | |
| --- | --- | --- | --- | --- |
|  | **Collaborative care**  **n=151** | **Consultation liaison n=151** | **Collaborative care n=196** | **Consultation liaison n=193** |
| Years of age, mean (95% CI) | 35 (33 to 37) | 36 (34 to 39) | 37 (35 to 39) | 40 (38 to 42) |
| Female, n (%) | 112 (74) | 98 (65) | 128 (65) | 123 (64) |
| **Primary diagnosis, n (%)** | | | | |
| Generalized anxiety disorder | 45 (30) | 45 (29) | - | - |
| Panic disorder/agoraphobia | 72 (48) | 71 (48) | - | - |
| Social anxiety disorder | 26 (17) | 26 (17) | - | - |
| Obsessive compulsive disorder | 8 (5) | 9 (6) | - | - |
| Mild depression | - | - | 16 (8) | 15 (8) |
| Moderate depression | - | - | 132 (67) | 130 (67) |
| Severe depression | - | - | 48 (25) | 48 (25) |
| **Secondary diagnosis, n (%)** | | | | |
| Anxiety diagnosis | 35 (23) | 52 (34) | 59 (30) | 68 (35) |
| Depression diagnosis | 22 (15) | 19 (13) | - | - |
| **Outcome measures, n (95% CI)** | | | | |
| BAI | 25.2 (23.8 to 26.6) | 25.4 (24.0 to 26.8) | 22.5 (21.0 to 24.1) | 21.6 (20.4 to 22.8) |
| BDI-II ^a^ | 22.2 (20.7 to 23.7) | 21.3 (19.6 to 22.9) | 32.1 (30.7 to 33.5) | 30.4 (29.3 to 31.5) |
| SDS ^b^ | 15.1 (14.0 to 16.2) | 15.0 (13.8 to 16.2) | 19.6 (18.7 to 20.5) | 19.2 (18.3 to 20.0) |
| Personal control ^c^ | 21.1 (20.4 to 21.7) | 20.5 (19.8 to 21.2) | 20.5 (20.0 to 21.1) | 20.7 (20.2 to 21.2) |
| Self-efficacy (obtain help) ^d^ | 6.4 (6.1 to 6.7) | 6.4 (6.1 to 6.8) | 5.4 (5.1 to 5.7) | 5.6 (5.3 to 5.8) |
| Self-efficacy (symptoms) ^e^ | 5.8 (5.5 to 6.0) | 5.8 (5.5 to 6.1) | 4.5 (4.3 to 4.8) | 4.7 (4.4 to 4.9) |
| WHO-5 ^f^ | 36.5 (33.5 to 39.4) | 38.2 (35.3 to 41.2) | 22.5 (20.3 to 24.6) | 23.9 (22.1 to 25.7) |
| SCL-90-R ^g^ | 113.4 (106.3 to 120.5) | 110.1 (102.5 to 117.6) | 129.5 (121.5 to 137.5) | 122.8 (116.8 to 128.7) |
| EQ-5D-3L | 0.67 (0.65 to 0.70) | 0.67 (0.64 to 0.70) | 0.62 (0.59 to 0.65) | 0.63 (0.61 to 0.66) |

Data in this table is reproduced from the article presenting 6-month follow-up results, which is distributed under the terms of the Creative Commons Attribution licence (http://creativecommons.org/ licenses/by/4.0/) (2)

Abbreviations: BAI: Beck Anxiety Inventory, BDI-II: Beck Depression Inventory-II, EQ-5D-3L: EuroQol Five Dimensions Questionnaire with Three Levels, SDS: Sheehan Disability Scale, SCL-90-R: Symptom Checklist-90-Revised, WHO-5: World Health Organization-5 Well-Being Index.

^a^ Missing data in the anxiety trial: 1 in the CL-group.

^b^ Missing data in the anxiety trial: 24 in the CC-group and 15 in the CL-group. Missing data in the depression trial: 29 in the CC-group and 25 in the CL-group.

^c^ Subscale Personal control from the Illness Perception Questionnaire-Revised (IPQ-R).

^d^ Subscale Obtain Help from Community, Family, Friends from the Chronic Disease Self-Efficacy Scales.

^e^ Subscale Control/Manage Depression from the Chronic Disease Self-Efficacy Scales.

^f^ Missing data in the depression trial: 1 in the CL-group.

^g^ Missing data in the anxiety trial: 1 in the CL-group. Missing data in the depression trial: 1 in the CL-group.

**Table S2a. Contacts between primary care providers and participants during 18 months in the Collabri Flex anxiety trial (N=302)**

**and the Collabri Flex depression trial (N=389)**

| **Depression trial** | | | | | | |
| --- | --- | --- | --- | --- | --- | --- |
|  | **Collaborative care** | | **Consultation liaison** | |  |  |
| **Contacts between providers and participants** | **N** | **R (95% CI)** | **N** | **R (95% CI)** | **RR (95% CI)** | ***P*** |
| GP, total contacts | 4726 | 24.1 (21.7, 26.7) | 4957 | 25.7 (23.2, 28.0) | 1.06 (0.93,1.18) | 0.282 |
| GP, talking therapy | 307 | 1.57 (1.29, 1.86) | 366 | 1.9 (1.56, 2.21) | 1.17 (0.89,1.49) | 0.190 |
| Private psychologist | 74 | 0.4 (0.2, 0.6) | 535 | 2.8 (2.1, 3.5) | 7.35 (4.05,19.97) | ≤0.001 |
| Private psychiatrist | 355 | 1.8 (1.0, 2.8) | 771 | 4.0 (1.8, 7.0) | 2.23 (0.92,5.23) | 0.115 |
| **Anxiety trial** | | | | | | |
|  | **Collaborative care** | | **Consultation liaison** | |  |  |
| **Contacts between providers and participants** | **N** | **R (95% CI)** | **N** | **R (95% CI)** | **RR (95% CI)** | ***P*** |
| GP, total contacts | 3754 | 24.9 (21.9, 28.0) | 3804 | 25.2 (22.6, 28.0) | 1.07 (0.93-1.22) | 0.264 |
| GP, talking therapy | 172 | 1.14 (0.82, 1.48) | 193 | 1.28 (0.97, 1.63) | 1.24 (0.84-1.87) | 0.208 |
| Private psychologist | 65 | 0.4 (0.2, 0.8) | 187 | 1.2 (0.7, 1.8) | 3.04 (1.37-9.52) | 0.022 |
| Private psychiatrist | 294 | 2.0 (0.9, 3.3) | 309 | 2.1 (1.1, 3.2) | 1.02 (0.46-2.71) | 0.494 |

Abbreviations. R: Rate, RR: Rate ratio. The rate is the number of events per person per 18 months. Note Collaborative care is the reference group when reporting RR. Note: Participants in the Collaborative care group, had on average 8.7 sessions (0-16) with their care managers, in both the Collabri Flex Depression Trial, and the Collabri Flex Anxiety trial. Information about contacts with private psychologists and psychiatrists is retrieved from the Public Health Insurance Register.

We do not have information about potential other contacts with private psychologists and psychiatrists.

**Table S2b. Proportion of participants having contacts with a private psychologist, private psychiatrist or having talking therapy with**

**a GP during 18-month follow-up in the Collabri Flex depression trial (N=389) and the Collabri Flex anxiety trial (N=302)**

| **Depression trial** | | | | |
| --- | --- | --- | --- | --- |
|  |  | **Collaborative care** | **Consultation liaison** |  |
|  | **Number of contacts** | **Number of persons, N (%)** | **Number of persons, N (%)** | ***P*** |
| GP talking therapy | 0 | 94 (48) | 72 (37.3) | 0.093 |
|  | 1-3 | 66 (33.7) | 82 (42.5) |  |
|  | 4+ | 36 (18.4) | 39 (20.2) |  |
| Private psychologist | 0 | 185 (94.4) | 135 (69.9) | ≤0.001 |
|  | 1+ | 11 (5.6) | 58 (30.1) |  |
| Private psychiatrist | 0 | 177 (90.3) | 164 (85) | 0.090 |
|  | 1+ | 19 (9.7) | 29 (15) |  |
| **Anxiety trial** | | | | |
|  |  | **Collaborative care** | **Consultation liaison** |  |
|  | **Number of contacts** | **Number of persons, N (%)** | **Number of persons, N (%)** | ***P*** |
| GP talking therapy | 0 | 96 (63.6) | 78 (51.7) | 0.010 |
|  | 1-3 | 33 (21.9) | 57 (37.7) |  |
|  | 4+ | 22 (14.6) | 16 (10.6) |  |
| Private psychologist | 0 | 141 (93.4) | 124 (82.1) | 0.003 |
|  | 1+ | 10 (6.6) | 27 (17.9) |  |
| Private psychiatrist | 0 | 137 (90.7) | 133 (88.1) | 0.436 |
|  | 1+ | 14 (9.3) | 18 (11.9) |  |

Abbreviations. CC: Collaborative care, CL: Consultation-liaison. GP: General practitioner.

Note: Participants in the Collaborative care group had on average 8.7 sessions (0-16) with their care managers, in both the Collabri Flex Depression Trial, and the Collabri Flex Anxiety trial. Information about contacts with private psychologists and psychiatrists is retrieved from the Public Health Insurance Register.

We do not have information about potential other contacts with private psychologists and psychiatrists.

**Table S3. Sensitivity analyses at 18-month follow-up in the Collabri Flex depression trial (N=389)**

**and the Collabri Flex anxiety trial (N=302)**

| **Depression trial** | | | | |
| --- | --- | --- | --- | --- |
|  | **Collaborative care** | **Consultation liaison** |  |  |
|  | **Mean BDI-II (95% CI)** | **Mean BDI-II (95% CI)** | ***P*** | **Cohen's d** |
| “Best-case” scenario analysis | 7.8 (6.6-9.0) | 9.5 (7.9-11.1) | 0.043 | 0.17 |
| “Worst-case” scenario analysis | 18.3 (16.7-19.9) | 22.0 (20.4-23.5) | 0.000 | 0.32 |
| Observed data | 11.5 (9.8-13.2) | 15.9 (13.5-18.3) | 0.000 | 0.40 |
| Adjustment for unequal baseline means | 11.5 (9.8-13.2) | 15.9 (13.5-18.3) | 0.000 | 0.37 |
| **Anxiety trial** | | | | |
|  | **Collaborative care** | **Consultation liaison** |  |  |
|  | **Mean BAI (95% CI)** | **Mean BAI (95% CI)** | ***P*** | **Cohen's d** |
| “Best-case” scenario analysis | 11.1 (9.8-12.5) | 9.1 (7.7-10.5) | 0.030 | 0.24 |
| “Worst-case” scenario analysis | 17.5 (16.2-18.9) | 20.3 (18.9-21.8) | 0.006 | 0.31 |
| Observed data | 14.1 (12.5-15.6) | 14.4 (12.1-16.8) | 0.450 | 0.04 |
| Adjustment for unequal baseline means | 14.0 (12.9-15.2) | 15.3 (13.9-16.7) | 0.177 | 0.16 |

Analyses are made for BAI in the anxiety trial and BD-II in the depression trial.

**Table S4. Subgroup analyses at 18-month follow-up in the Collabri Flex depression trial (N=389)**

**and the Collabri Flex anxiety trial (N=302)**

| **Depression trial** | | | | | | | |
| --- | --- | --- | --- | --- | --- | --- | --- |
|  |  | **Collaborative care** |  | **Consultation-liaison** |  |  |  |
|  | **N** | **Mean BDI-II (95% CI)** | **N** | **Mean BDI-II (95% CI)** | **Est (95% CI)** | ***P*** | **Cohen's d** |
| **Depression severity** | | | | | | | |
| Mild depression | 16 | 11.1 (6.9-15.3) | 15 | 12.9 (8.8-16.9) | -0.36 (-4.97-4.25) | 0.879 | 0.23 |
| Moderate depression | 132 | 11.9 (10.5-13.3) | 130 | 15.4 (13.7-17.2) | 3.96 (2.03-5.88) | 0.000 | 0.38 |
| Severe depression | 48 | 13.8 (11.3-16.4) | 48 | 17.6 (14.2-21.0) | 7.86 (3.80-11.92) | 0.000 | 0.36 |
| **Previous/current treatment** | | | | | | | |
| Previous/current treatment | 82 | 12.5 (10.7-14.3) | 81 | 15.9 (13.8-18.0) | 4.96 (2.50-7.42) | 0.000 | 0.38 |
| No previous/current treatment | 114 | 12.2 (10.7-13.7) | 112 | 15.7 (13.7-17.7) | 4.01 (1.81-6.21) | 0.000 | 0.36 |
| **Personality disorder screening** | | | | | | | |
| Positive screening, SAPAS>2 | 59 | 13.3 (11.0-15.6) | 67 | 16.2 (13.7-18.8) | 5.36 (2.20-8.53) | 0.001 | 0.30 |
| No positive screening, SAPAS≤2 | 137 | 11.2 (9.8-12.6) | 126 | 15.6 (13.6-17.5) | 4.35 (2.33-6.36) | 0.000 | 0.48 |
| **Anxiety trial** | | | | | | | |
|  |  | **Collaborative care** |  | **Consultation liaison** |  |  |  |
|  | **N** | **Mean BAI (95% CI)** | **N** | **Mean BAI (95% CI)** | **Est (95% CI)** | ***P*** | **Cohen's d** |
| **Primary anxiety diagnosis** | | | | | | | |
| Generalized anxiety disorder | 45 | 12.4 (10.4-14.3) | 45 | 14.4 (11.6-17.2) | 0.73 (-2.19-3.64) | 0.627 | 0.25 |
| Panic disorder/agoraphobia | 72 | 14.3 (12.5-16.0) | 71 | 15.3 (13.2-17.3) | 1.43 (-0.88-3.73) | 0.228 | 0.12 |
| Social anxiety disorder | 26 | 15.5 (12.6-18.3) | 26 | 17.4 (13.9-20.9) | 1.81 (-1.52-5.14) | 0.292 | 0.24 |
| Obsessive-compulsive disorder | 8 | 16.6 (8.4-24.9) | 9 | 14.6 (12.0-17.2) | -0.30 (-4.24-3.65) | 0.885 | 0.30 |
| **Previous/current treatment** | | | | | | | |
| Previous/current treatment | 73 | 14.1 (12.3-16.0) | 71 | 16.9 (14.5-19.3) | 1.95 (-0.59-4.50) | 0.135 | 0.30 |
| No previous/current treatment | 78 | 14.0 (12.4-15.5) | 80 | 13.9 (12.4-15.4) | 0.46 (-1.30-2.22) | 0.611 | 0.01 |
| **Personality disorder screening** | | | | | | | |
| Positive screening, SAPAS>2 | 49 | 15.4 (13.2-17.3) | 56 | 16.5 (14.1-18.9) | 0.56 (-2.05-3.17) | 0.676 | 0.15 |
| No positive screening, SAPAS≤2 | 102 | 13.5 (11.9-14.8) | 95 | 14.2 (12.4-16.1) | 1.55 (-0.44-3.55) | 0.129 | 0.11 |

References

1. Hesse M, Moran P. Screening for personality disorder with the Standardised Assessment of Personality: Abbreviated Scale (SAPAS): further evidence of concurrent validity. BMC Psychiatry. 2010 Jan;10:10.

2. Curth NK, Hjorthøj C, Brinck-Claussen U, Jørgensen KB, Rosendal S, Bojesen AB, et al. The effects of collaborative care versus consultation liaison for anxiety disorders and depression in Denmark: two randomised controlled trials. Br J psychiatry [Internet]. 2023;1–8. Available from: http://www.ncbi.nlm.nih.gov/pubmed/37395101
